# Research on the Quality of Partially Removable Skeletal Prostheses Made Using Classical Versus Modern Sintering Techniques

**DOI:** 10.3390/biomedicines11092397

**Published:** 2023-08-27

**Authors:** Magda-Ecaterina Antohe, Cristina Gena Dascălu, Doriana Agop Forna, Elena Gabriela Hitruc, Nicanor Cimpoeșu, Norina Consuela Forna

**Affiliations:** 1 3rd Dental Medicine Department, Faculty of Dental Medicine, “Grigore T. Popa” University of Medicine and Pharmacy, 700115 Iasi, Romania; magda.antohe@umfiasi.ro (M.-E.A.); norina.forna@umfiasi.ro (N.C.F.); 2 Department of Medical Informatics, “Grigore T. Popa” University of Medicine and Pharmacy, 16 Universității Street, 700115 Iasi, Romania; 3 1st Dental Medicine Department, Faculty of Dental Medicine, “Grigore T. Popa” University of Medicine and Pharmacy, 16 Universității Street, 700115 Iasi, Romania; 4 “Petru Poni” Institute of Macromolecular Chemistry, Aleea Grigore Ghica-Vodă, 41A, 700487 Iasi, Romania; gabihit@icmpp.ro; 5 Faculty of Materials Science and Engineering, “Gheorghe Asachi” Technical University, Bulevardul Profesor Dimitrie Mangeron 67, 700050 Iasi, Romania; nicanor.cimpoesu@academic.tuiasi.ro

**Keywords:** partially removable skeletal dentures, casting technique, digital technologies, alloys

## Abstract

Conventional partially removable skeletal dentures are one of the most common therapeutic solutions offered to edentulous patients worldwide. The present study aims to compare the skeleton of removable dentures realized via classical techniques to that realized via modern techniques, represented by the laser sintering technique, with the comparative aspects being realized through the evaluation of atomic force microscopy (AFM). A total of 20 metal frameworks made of Co-Cr were sectioned, representing the infrastructure of partially removable skeletal dentures, developed using the classical technique versus the laser sintering technique. The infrastructures of partially removable skeletal dentures were designed for both the maxilla and the mandible, with the design of each type of denture being identical, and were developed using both techniques. The roughness values are different depending on the technological method used; for the conventional casting technique, we have higher roughness for the component elements of the partially removable skeletal denture that have more stretch, e.g., the major connector, and for the 3D laser sintering technique, lower roughness is obtained for the component elements that have a lower stretch, e.g., the clasp arms, the minor connector, or the junction between the saddles and the major connector. The clinical implications of the presence of roughness at the level of the active arms or at the level of the connector saddle junction are represented by the risk of fracture, which confers real discomfort to the patient.

## 1. Introduction

A partially removable skeletal denture is a complex therapeutic solution with numerous advantages in the rehabilitation of the morphology and functions affected by edentulousness [1].

Conventional partially removable skeletal dentures are one of the most common therapeutic solutions offered to edentulous patients worldwide [2,3].

In order to ensure the success of oral rehabilitation treatment, it is necessary to understand both the needs of the patients and the clinical and technological challenges faced by the medical team [4].

The success of a prosthetic treatment using partially removable skeletal dentures is correlated with the observance of the particularities of the clinical case. Individualization of the design of this type of denture is carried out according to known therapeutic principles, in accordance with the biomaterials and technology used [5].

The balance between biomechanical and biological requirements leads to long-term maintenance of the oral structures to which the partially removable skeletal denture is applied [6].

The biomechanical principles proposed by McCracken have important clinical implications, ensuring very good retention and stability of the prosthetic construction through the uniform distribution of forces in the supporting areas [7].

Marxkors paid particular attention to the realization of so-called hygienic design, based on biological principles, in which the marginal gingiva is free so that the final shape of the designed denture allows for dental plaque control to prevent caries and periodontal disease [8,9].

In the context of the impressive prosthetic rehabilitations offered by oral implantology and implant prosthodontics, in conjunction with current biomaterials and CAD-CAM technology, partially removable dentures remain a viable treatment alternative for an important category of patients with low incomes [10,11].

Achieving a partially removable skeletal denture at optimal parameters is based on elective diagnosis, rigorous planning, and monitoring. Among the factors involved in the final therapeutic success of a partially removable skeletal denture, we can mention impeccable adaptation of the denture infrastructure and the correlation of the edentulous topography with the design of the mechanical skeleton, while special attention must be paid to communication with the dental laboratory as well as the practical results of patient education on the care and maintenance of this type of denture [12,13].

Contemporary medical practice confronts us with two types of prostheses from the point of view of technological realization: a first type belonging to the classical techniques and a second type belonging to the digital techniques [14,15].

The technological algorithm for making partially removable skeletal dentures involves a casting technique, while current digital technologies allow for the design of skeletal denture components via 3D representation instead of casting, using analysis tools that create precision prosthetic components at the micrometer level [16,17].

CAD-CAM systems allow the frameworks of partially removable restorations to be fabricated without the use of wax or the classic alloy casting process, leading to maximum efficiency, precision, and certain time-related economic benefits [18].

Partially removable dentures (PRDs), due to their conservative nature, influence, in a defining way, the quality of life of many partially edentulous patients around the world; thus, more than 13% of adult patients in North America and Europe wear these types of prosthetic constructions. The metal framework of partially removable skeletal dentures can conventionally be made of cast alloys via a laborious technological algorithm, subject to human error, that is based on the lost-wax technique. The four basic components involved in PRDs are the artificial teeth, the denture base, retainers, and connectors [19].

In line with the digitization process that governs contemporary dentistry and in the field of partially removable skeletal prosthetics, PRD frameworks using digital rapid prototyping techniques have recently been introduced. Rapid prototyping additive manufacturing technologies include stereolithography, selective laser melting, selective laser sintering, selective deposition modeling, 3D printing, and direct inkjet printing [20].

Stereolithography is known in the Technology Register as the first prototyping technique introduced on the market and was the first technique used in the early 2000s in the fabrication of the framework of partially mobilizable skeletal dentures [21].

The year 2006 represents the introduction of the selective laser melting technique for the direct fabrication of the metal framework of computer-designed skeletal dentures, a very important step in technological evolution resulting in the elimination of the conventional casting process. The first software (Tang Long CAD), developed specifically for designing PRD frameworks for rapid prototyping, was released in 2010 [22].

Today, laser sintering technologies are becoming more and more popular due to the precision of the metal framework, reducing the manual machining errors found in conventional techniques, resulting in optimal quality. It is important to mention that the current laser sintering technology for the skeleton of partially mobile skeletal dentures has its limitations, linked, on the one hand, to the costs of the equipment and software, and on the other hand, to the particularity of the clinical case, which must contend with the limitations of the available software [23,24].

In addition to the technological line addressed, a particularly important role is played by the biomaterials chosen in contemporary practice, with Cr-cobalt and titanium being the materials most often used in the realization of the framework of partially removable skeletal dentures.

## 2. Purpose of the Study

The present study aims to compare the infrastructure of removable dentures realized via classical techniques to that realized via modern techniques, represented by the 3D laser sintering technique, with the comparative aspects being realized through the evaluation of atomic force microscopy (AFM).

## 3. Materials and Methods

**Figure 1:**
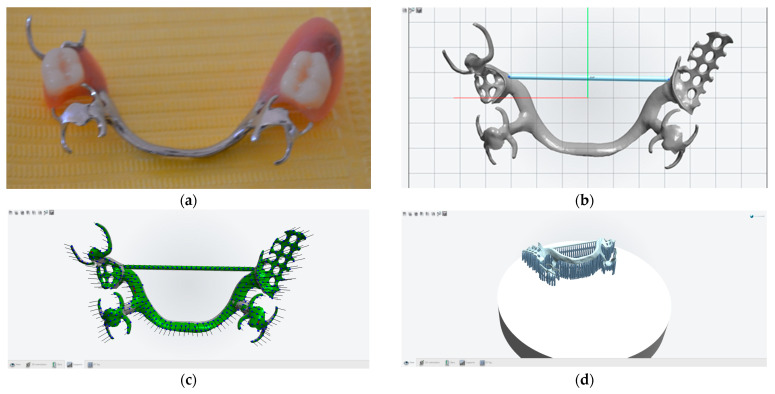
Partially removable skeletal dentures: classical approach (**a**) and the laser sintering technique (**b**–**d**).

A total of 20 metal frameworks made of Co-Cr were sectioned, representing the infrastructure of partially removable skeletal dentures, developed via the classical technique versus the 3D laser sintering technique. The infrastructures of the partially removable skeletal dentures were designed for both the maxilla and the mandible, with the design of each type of denture being identical, and were developed using both techniques (Figure 1). They were then subjected to AFM techniques; sections were made of each component element of the partially removable skeletal denture, as well as the essential points of connection between them, so that a comparative analysis between the two techniques could be made.

In conventional technology, alloy casting is performed using Trisolite equipment, whereby the alloy is melted in the crucible by an induction current in a controlled environment, with the application of argon-type inert gases and casting automatically triggered when the melting range is reached.

The Cr-Co alloy used is specially designed for skeletal dentures and has high strength. Melting is carried out directly in the crucible and is introduced when it is completely melted. For the modern technique, sintering was performed at 800 degrees Celsius for 35 min, and the digital design was created using the EXOCAD system.

In the case of the analysis of metal elements for dental applications, an NTEGRA scanning probe microscope (NT-MDT Spectrum Instruments, Moscow, Russia) in AFM configuration was used. The surface images were obtained using a Solver PRO-M scanning probe microscope (NT-MDT, Spectrum Instruments, Moscow, Russia), in AFM configuration. Rectangular silicon cantilevers NSG10 (NT-MDT, Spectrum Instruments, Moscow, Russia) with tips with a high aspect ratio (sharpened pyramidal tip, angle of nearly 20°, tip curvature radius of 10 nm, and height of 14–16 µm) were used in order to minimize convolution effects. All images were acquired in air, at room temperature (23 °C), in tapping mode, with a velocity of 6 mm/s. For image acquisition, Nova v.19891 Solver software was used [25].

The null hypothesis is based on the idea that the surface roughness of partially removable skeletal dentures made using the digital technique and the classical casting technique have similar values.

## 4. Results

**Figure 2:**
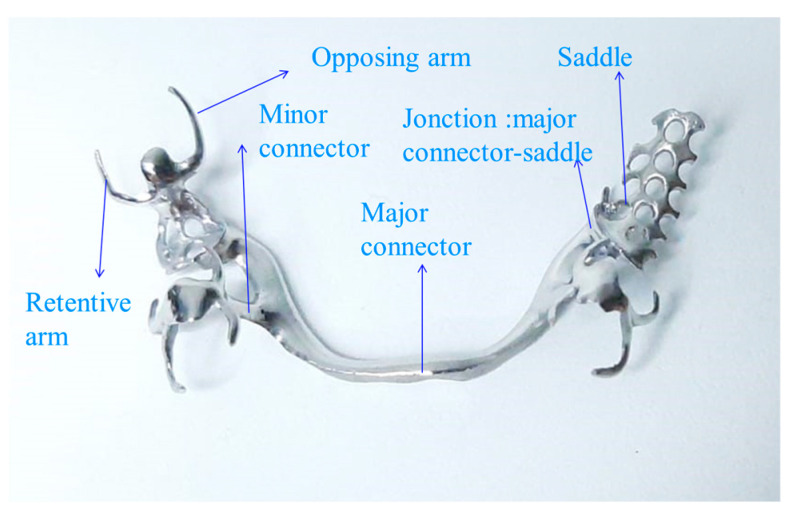
The component elements of the PRDs, analyzed following AFM.

The metal frameworks of the partially removable skeletal dentures analyzed, made of Co-Cr alloy, were obtained using two different methods: classical casting and 3D laser sintering (Figure 2).

The aim was to identify and analyze the surface condition of the specimens after the processing stage via atomic force microscopy (AFM), a technique confirmed in the analysis of materials and elements with medical applications [26].

**Figure 3:**
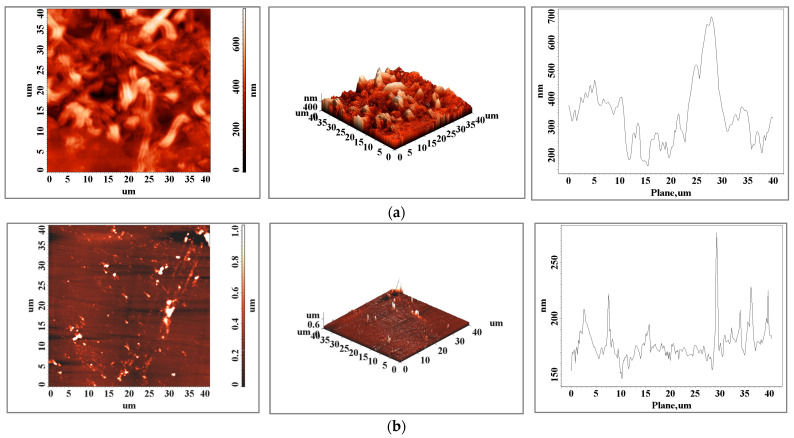
Atomic force microscopy images of the surface of a (**a**) cast and (**b**) 3D-printed main connector element for an area of 40 × 40 μm^2^.

Figure 3 shows the 2D and 3D surface details of the main connector element made using two different processing techniques: classical casting in (a) and 3D laser sintering in (b). The 2D and 3D scans show a surface with higher roughness in the case of the casting sample (Figure 3a). The roughness differences obtained at different scan scales (10 × 10 versus 60 × 60) show larger variation in roughness on the surface of the classic cast sample, with a roughness ratio of 0.65 versus 0.37 for the 3D laser-printed sample.

Comparing the two samples at the 10 × 10 μm scale, the roughness ratio for this element is 4.9, while for the 60 × 60 μm scale, it is 2.8, which shows that large variations between the two samples occur at the micrometric level.

The surface roughness state of the elements, obtained via atomic force microscopy scans, present a variation between small area values and bigger ones that can influence, at the micrometric scale, the metallic elements’ resistance to fatigue and can influence the lifetime of the materials [27]. The topography of the cast CoCr alloy (Figure 3a) shows the typical interdendritic structures of the cast alloy, identified as eutectic “self-blocked carbides” of the M23C6 secondary phase type [28,29].

**Figure 4:**
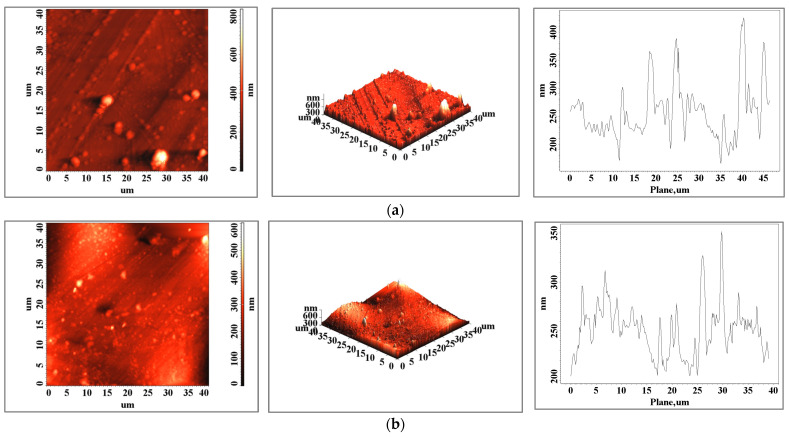
Surface images of a (**a**) cast and (**b**) 3D-printed dental segment bracket arm element made via atomic force microscopy for an area of 40 × 40 μm^2^.

**Figure 5:**
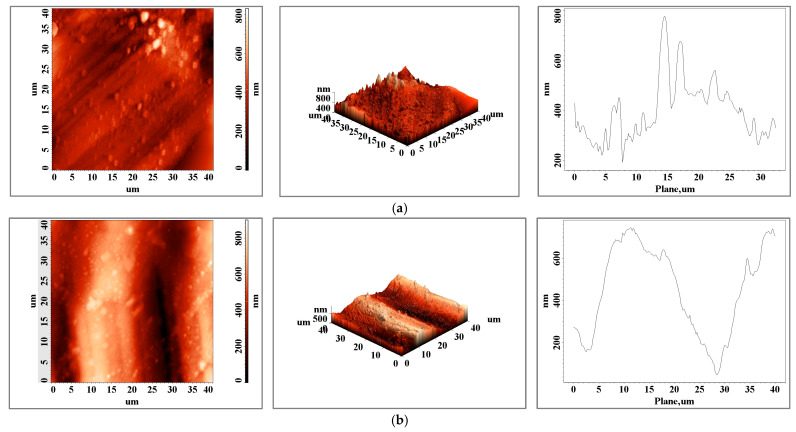
Atomic force microscopy images of the surface of a (**a**) cast and (**b**) 3D-printed sea mesh element for an area of 40 × 40 μm^2^.

The relief distribution plots show a more homogeneous surface when the sample is obtained via additive manufacturing and one of the subsequent surface preparation steps can be eliminated. In the case of the bracket arm element, the surfaces are very close in terms of roughness (Table 1 and Figure 3), both in terms of values and the distribution of peaks on the surface. For this element, we can consider both techniques viable technologies with similar results in this case. The surface topography of the 3D-printed sample shows a textured surface (Figure 4b, both 2D and 3D scans) with very fine cellular dendrites caused by the rapid cooling rate during the solidification process [30]. In both the classically cast and 3D-printed elements, the presence of solidified material particles (Figure 3, Figure 4 and Figure 5) with sizes of a few micrometers is observed on the surface.

**Figure 6:**
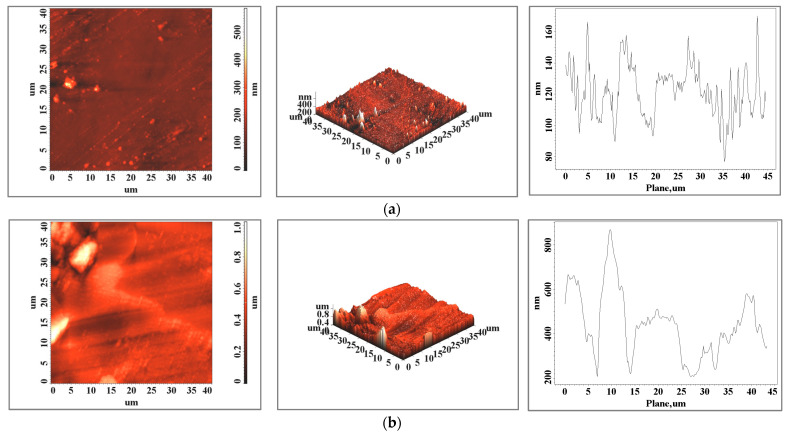
Surface images of a (**a**) cast and (**b**) 3D-printed opposing arm element made using atomic force microscopy for an area of 40 × 40 μm^2^.

In the case of the opposing arms of the clasps, a surface with a higher roughness is obtained via 3D printing due to the size of this element, the geometry, and the alignment of the support network (Figure 6).

For the opposing arm element, the roughness of the 3D-printed sample surface is higher and the difference between the values increases for larger areas (probably because of the surface structural details or 3D printing network traces) from a roughness value of 3.31 for a 10 × 10 area to 7.76 for a 60 × 60 area.

## 5. Discussion

Understanding how the microstructure of a material is affected by the manufacturing process can provide insight into why printed parts produced via conventional casting or additive manufacturing sometimes fail. AFM is a technique that can characterize the surface topography of materials with a nanoscale resolution. AFM uses an ultra-sharp tip to touch and “feel” the surface of a material, mapping the topography line-by-line in a raster scan pattern. Unlike electron microscopy (SEM), AFM generates true 3D quantitative topography data, works equally well on both conductive and insulating samples, and does not require thin sections or staining. Compared to optical microscopy or profilometry, AFM is not limited by the optical diffraction limit, so newer AFMs can easily image with nanoscale lateral resolution and sub-Angstrom for vertical resolution.

All the components of a partially removable skeletal denture (PRD) contribute in a definitive way to the integration of the denture into the harmony of the somatogenic system, leading to optimal morphological and functional restoration.

The final design of partially removable skeletal dentures differs from case to case, and the main connector that has the greatest stretch differs depending on the architecture of the prosthetic field, so that the final shape has very good biomechanical balance.

The supporting and stabilizing elements represented by the clasps are also of different types and the choice of which to use is made in accordance with biomechanical and biological principles. The results of our study indicate that the size of each component element of the partially removable skeletal denture influences the final roughness depending on the technology used—conventional or 3D laser sintering. At the level of the main connector, the component with the largest extension within the partially removable skeletal denture, we note at the 10 × 10 scale a roughness of 70, compared to a roughness of 14 for the prosthetic part made via sintering technology. The differences in roughness in terms of the active arm of the clasp, which is the element that maintains support and stabilizes the prosthesis, are relatively close to 29 for the classic casting technique and 20 for the modern technique represented by laser sintering.

The higher roughness present on the active arm of the clasps created using the casting technique influences the biomechanical behavior of the denture; in daily practice, most types of fractures occur at this level following insertion and disinsertion of the denture; therefore, the risk of fracture is correlated with the surface roughness and the degree of fatigue of the alloy.

The fracture of the component elements of the metallic infrastructure of a partially removable skeletal denture is based on an accumulation of factors, of which we can mention alloy fatigue, and structural defects such as porosities, inclusions, and surface defects [31].

Thus, alloy fatigue occurs as a consequence of the loss of mechanical properties under repetitive applied forces [32].

Frequently located at the level of areas with structural imperfection, quantified by the degree of roughness, it is important to note that alloy fatigue can also occur at points where high stresses are concentrated. Thus, fractures caused by fatigue during insertion and disinsertion of the denture are found in the arms of the brackets, in addition to surface defects. In relatively rare cases, fractures of the main connectors are mentioned in the literature; in these cases, alloy fatigue is correlated with undersizing of this element of the prosthetic part. Fractures of the bracket arms can be explained by changes in alloy properties, such as tensile strength, due to errors in casting techniques [13].

With regard to changes in the denture saddle, a component element of the metallic infrastructure of the partially removable skeletonized denture, we note a higher value of roughness for the 3D laser sintering technology and a lower value for the classical technique. These differences related to the technological characteristics are important in dental medicine because fractures can occur at the junction between the saddle and the connector in the context of different static and dynamic mandibular occlusion characteristics.

The results of our study are related to the results of other studies that have addressed the same topic. Y.S. Al Jabbari et al. in 2014 evaluated the porosity, microstructure, and Vickers hardness of different types of specimens using a classical technique compared to the laser sintering technique.

The difference in the design methodology of our study lay in the fact that complete metal skeleton structures were developed, maintaining the same design in both the classical and digital techniques. The results obtained by the authors indicated the absence of porosities in specimens made via the digital technique compared to their presence in the classical technique that used casting. The digital specimens also obtained the best results in terms of hardness compared to the classical technique [33].

Studies by Takaichi et al., in 2013, using a cobalt–chromium–molybdenum alloy, aimed to evaluate the biomechanical properties of the alloy produced using the SLM technique compared to the classical technique, obtaining optimal strength parameters compared to the alloy cast via the classical technique [34]. Qian and co-workers, in their research conducted in 2015, conducted an investigation of the characteristics of alloys involved in the structure of skeletal protein parts associated with digital and classical techniques; through an analysis from the nano- to the macro-level, they found that although defects were detected, the yield and strength elements were much higher using the digital technique compared to cast alloy [35].

The differences obtained in the results of our study provide data on the similarity in the roughness present in some component elements of skeletal dentures, such as the active arm of the Ackers bracket, which was similar in both techniques; different results were obtained for the other component elements, such as the main connectors, where superiority was found in the digital technique [32,36,37,38,39,40].

In our study, at the level of the metal infrastructure of the saddles, higher porosity was observed for the digital technique, which is associated with the design presenting successive holes of the metal elements that materialize as retentions for the future acrylic component.

The laser sintering technique produces a precise digitally designed model, resulting in a denture that is rigorously adapted to the specific features of each clinical case [32,37,38,39].

The different results obtained in terms of roughness at the level of the component elements of the partially removable skeletal denture infrastructure made using the digital technique compared to the classical technique showed different values at the level of certain components, which practically cancels the null hypothesis [41,42,43,44,45,46].

Current trends in dental prosthetics are moving towards the quantifiable implementation of digital strategies in the therapeutic sphere of partially removable skeletal prosthetic applications, leading to optimal long-term outcomes as a result of improved planning and design techniques in conjunction with new and efficiently improved biomaterial structures, further leading to a definite increase in the quality of life of patients [47,48,49,50].

The limitations of this study are related to the fact that the research was conducted in vitro, in which the behaviour of the dentures differs from the biological and biomechanical behavior of a partially removable skeletal denture in a patient’s oral cavity under the pressure of masticatory forces in the context of different types of static and dynamic occlusion. Such an in vivo study requires a long evaluation period to see how the particularities of the clinical case influence the structure of partially removable skeletal dentures and the risk of fracture.

Similarly, as a future research direction, it is necessary to make different alloys and compare the final behavior of the skeletal dentures.

## 6. Conclusions

The roughness of the metal framework of partially removable skeletal dentures is different depending on the technologies used, i.e., conventional casting or laser sintering techniques.

The sizes of the component parts of removable skeletal dentures made using these two techniques influence the roughness of their surfaces.

Higher roughness is present at the level of the main connectors, with a roughness difference compared to the classical technique of 4.9 for the 10 × 10 scale and 2.8 for the 60 × 60 scale, which is found at the micrometric level.

For the component elements of the small-scale skeletal denture infrastructure, a conclusive example being the active arm of the clasp, the roughness is higher for the laser sintering technique, with a roughness of 29 for the cast elements and 20 for the laser sintering technique at the 10 × 10 scale.

The clinical implications of the presence of roughness at the level of the active arms or at the level of the connector saddle junction are represented by the risk of fracture, which confers real discomfort to the patient.

The structure is dendritic in both cases but, for additive manufacturing, it is much finer, primarily due to the higher solidification speed following local laser melting.

Both techniques require subsequent heat treatments, but by selecting the right technique, surface preparation steps can be eliminated prior to the practical application of the elements.

The superiority of digital solutions in the production of partially removable skeletal dentures compared to traditional techniques leads to extremely precise therapeutic solutions with optimal integration at the level of the functionality of the dental system, creating certain premises for increasing the quality of life of patients wearing this type of denture.

## Figures and Tables

**Table 1:** Roughness values taken from the surfaces of the investigated elements.

	10 × 10	20 × 20	40 × 40	60 × 60
**Classically cast elements**				
21_211_MAJOR CONNECTOR (A)	70.37	105.86	112.80	107.91
21_212_MINOR CONNECTOR (A)	18.41	36.32	47.67	----
21_213_ RETENTIVE ARM OF THE CLASPS (A)	29.98	30.76	56.63	85.47
21_214_OPPOSING ARM OF THE CLASPS (A)	8.85	13.91	24.58	29.48
21_215_SADDLE (A)	26.30	40.37	81.73	113.41
**3D-printed elements (laser)**				
21_216_MAIN CONNECTOR (B)	14.40	16.51	31.77	38.35
21_217_SADDLE (B)	28.34	66.86	191.41	192.09
21_218_BRACKET ARM (B)	20.09	40.78	62.01	----
21_219_ OPPOSING ARM OF THE CLASPS (B)	29.38	65.72	132.76	228.96
21_220_MAJOR CONNECTOR JUNCTION WITH SADDLE (B)	7.89	12.79	19.30	175.04

## Data Availability

The data presented in this study are available on request from the corresponding author.
